# Reading fluency and word segmentation agreement modulate the benefits of word boundary cues for older readers in traditional Chinese

**DOI:** 10.3758/s13423-026-02884-w

**Published:** 2026-03-11

**Authors:** Yiu-Kei Tsang, Ming Yan, Jinger Pan

**Affiliations:** 1 Department of Education and Psychology, Hong Kong Baptist University, Kowloon, Hong Kong; 2 Department of Psychology, University of Macau, Taipa, Macau; 3 Center for Cognitive and Brain Sciences, University of Macau, Taipa, Macau; 4 Department of Psychology, The Education University of Hong Kong, New Territories, Hong Kong

**Keywords:** Cognitive aging, Reading, Eye movement, Chinese, Word boundary

## Abstract

Despite having extensive reading experience, older readers suffer from declines in visual acuity and processing speed, which may undermine their ability to segment words in unspaced scripts like Chinese. While using text colors to highlight word boundaries can aid reading and eye movement control for readers of simplified Chinese, it remains unclear whether the benefits extend to older readers, especially those who read the visually more complex traditional Chinese script. This study investigated this question in three conditions: a baseline monocolor condition, a word segmentation condition where words were marked by alternating text colors, and a nonword segmentation condition. By tracking the eye movements of 76 older readers, we found a robust interference effect from nonword segmentation across all reading and oculomotor measures. In contrast, benefits of word segmentation cues were strikingly specific, emerging only for readers with lower vocabulary knowledge and for words with clear, unambiguous boundaries. This reveals that the utility of explicit word boundary cues depends on a dynamic interplay between visual processing and vocabulary knowledge. These findings have important implications. Theoretically, they underscore that models of reading should account for word boundary ambiguity and readers’ experience. Practically, the development of assistive reading technologies needs to be tailored to the needs of less proficient readers, who benefit most from external support.

## Introduction

Research of reading among older adults has gained momentum in the last decade. Older readers experience declines in processing speed and visual acuity, yet they possess a wealth of lifetime reading experience, leading to compensatory reading strategy (Rayner et al., 2009). Understanding how these factors interplay is essential for developing age-sensitive reading models and designing effective interventions. Models of eye-movement control in reading, such as E-Z Reader (Reichle et al., 2003) and SWIFT (Engbert et al., 2005), posit words as fundamental units that guide saccades and fixations. In many alphabetic scripts, physical spaces demarcate word boundaries, making them perceptually salient. This is not the case for unspaced scripts like Chinese, where the concept of word is fuzzy and disagreement in word segmentation is common (Hoosain, 1992; Tsang et al., 2025). Nevertheless, word remains a crucial unit in Chinese reading, as many characters are homonymous (e.g., “教” can mean “to teach” or “religion”) and require the word context for disambiguation (Pan et al., 2023; Tsang & Chen, 2013). Furthermore, empirical corpus studies have consistently shown that word-level properties significantly influence eye movements during Chinese reading (Li et al., 2014; Pan et al., 2022; Yan et al., 2025). These findings suggest that Chinese readers are equipped with mechanisms to segment words in continuous text (Hoosain, 1992). This raises the questions of how the extensive reading experience and reduced visual acuity in older readers may modulate word segmentation and whether providing explicit word boundary cues would enhance reading performance.

Due to the absence of inter-word spacing in written Chinese, whether eye movements are influenced by word boundaries has been a longstanding topic of debate. Yan et al. (2010) proposed a flexible saccade planning model that assumes word segmentation begins early in the parafovea. If readers can successfully estimate boundaries of the upcoming word, they will target its center for optimal processing. Otherwise, the saccade target will be the beginning of the next word to obtain more information for segmentation. A subsequent study by Yen et al. (2012) proposed that proficient Chinese readers rely on basic statistical patterns—such as the likelihood of a character occurring in specific positions within a word—to compensate for the absence of interword spacing in Chinese text. Studies on Thai, another unspaced language, have supported the role of parafoveal statistical cues in word segmentation (Kasisopa et al., 2013; Reilly et al., 2005). Fan and Reilly (2022) proposed a similar idea, suggesting short and long saccades as word driven and character driven, respectively, based on the idea that readers cannot obtain enough segmentation cues parafoveally when a saccade is launched far away. Similarly, in the latest version of Chinese E-Z Reader model (Liu et al., 2025), word segmentation begins as the system considers the familiarity of different possible character groupings in the parafovea, which depends on factors like word frequency and orthographic neighborhood size. Others proposed that word segmentation occurs relatively late (Liu et al., 2015). According to this view, all characters within the perceptual span are processed in parallel, activating multiple word candidates simultaneously. A word is segmented from the character string when it surpasses an activation threshold and is recognized. In other words, word segmentation is effectively the by-product of word recognition, and any factors that help recognition will facilitate segmentation.

These different views all predict that providing explicit boundary cues should facilitate Chinese reading because it simplifies the process of word segmentation. Hsu and Huang (2000) showed that inserting spaces between words increased reading speed. Similarly, Bai et al. (2013) and Blythe et al. (2012) showed that word spacing facilitated new word learning and reading in children, presumably because the characters bind more tightly as a word. In contrast, Bai et al. (2008) observed no facilitation in reading speed in adult readers by word spacing, but reading was impeded when spaces were inserted after every character or when they grouped characters into nonwords.

An alternative method, color-based grouping, can provide boundary cues while retaining the original physical length. Perea and Wang (2017) found that marking word boundary with color led to faster oral reading for difficult texts. Similarly, Zhou et al. (2018) used alternating colors to highlight either words or nonwords. Global reading speed was slower with nonword grouping but comparable with normal sentence with word grouping. Critically, when word boundaries were marked, readers made fewer refixation and were more likely to fixate near words’ centers. Subsequent research with color cues has demonstrated similar word segmentation benefits in developing and L2 readers (Pan et al., 2021; Zhou et al., 2020). The available evidence thus supports facilitation by explicit word boundary cues, especially in disadvantaged readers like children and L2 learners.

Recently, Pan et al. (2025) applied the color-based boundary cue paradigm to older readers of simplified Chinese. Despite slower reading overall, older adults exhibited stronger interference from nonword segmentation and greater benefits from word segmentation. This increased sensitivity to explicit word boundary cues may reflect their reduced visual acuity, making word segmentation particularly challenging without additional perceptual support. However, their reading materials (i.e., the Beijing Sentence Corpus; Pan et al., 2022) featured minimal word boundary ambiguity. Therefore, whether the findings can be generalized to typical Chinese texts, where word boundaries are often unclear, requires further investigation. In addition, it remains to be tested whether individual differences may modulate the word boundary effects.

This study aims to replicate and extend the findings of Pan et al. (2025) along three dimensions. Firstly, we test the effects of segmentation cues in traditional Chinese. Although the simplified and traditional scripts are semantically and syntactically similar, traditional characters are visually more complex. Research in both isolated word recognition (Tsang et al., 2024) and sentence reading (Yan et al., 2025) has revealed subtle differences in processing the two scripts. Moreover, some traditional characters, like 臺 (platform), 檯 (desk), and 颱 (typhoon), are simplified into a single character (台). This may complicate word segmentation because each simplified character can form more words. It is an empirical question whether providing segmentation cues is particularly helpful for older readers of traditional Chinese.

Secondly, this study examines how individual differences in vocabulary knowledge may modulate the effectiveness of segmentation cues among older readers. Word boundary marking facilitated reading more robustly in children than adults (Bai et al., 2008; Blythe et al., 2012), suggesting that the benefits from explicit word boundary cues may vary depending on language skills. Although older readers as a group have accumulated more reading experiences than young readers, individual differences also exist. As aforementioned, without explicit cues, word segmentation relies on lexical and statistical information. Then, it is plausible that older readers with low vocabulary knowledge will have less knowledge for segmentation and benefit more from explicit visual cues. Alternatively, one may argue that those with poor lexical quality will read words analytically based on characters rather than holistic words (Chu & Leung, 2005). Then, word boundary cues may be irrelevant to their reading, leading to small or no benefits of segmentation cues. By testing these opposing views, this study will shed light on the interplay between perceptual and experience-related factors in older readers.

Finally, we examine whether a word’s segmentation agreement modulates the effects of segmentation cues. Pan et al. (2025) deliberately used words with minimal segmentation disagreement, which, while ensuring the validity of the experimental manipulation, prevented an analysis of this modulation. Segmentation ambiguity is a common phenomenon in Chinese (Tsang et al., 2025), and it can influence eye-movement control in reading (e.g., Ma et al., 2014). For instance, character strings with word boundary ambiguities not only increase fixation duration (Inhoff & Wu, 2005), but also modulate saccadic planning (Yan & Kliegl, 2016). In this study, items with a larger variation in segmentation agreement were used to explore how this factor interacts with the effects of segmentation cues. Two opposing possibilities are considered: Words with low boundary agreement are difficult to segment, making them benefit more from explicit segmentation cues. Alternatively, a low agreement implies that readers tend to parse a character string in multiple ways. An externally imposed boundary may therefore mismatch with some readers’ personal parsing, “diluting” the benefits of boundary cues.

## Method

### Participants

Seventy-eight older adults were recruited from the local Hong Kong community through advertisements at community centers, online forums, and referrals. Two participants were excluded from the analysis due to low comprehension accuracy (<70%) in the sentence reading experiment, resulting in a final sample of 76 (40 women). This sample size is comparable to or larger than those in previous studies on the effects of word segmentation cues in Chinese reading (e.g., Pan et al., 2025; Zhou et al., 2018) and adheres to a recent recommendation by Kumle et al. (2021). All participants were native Cantonese speakers who used traditional Chinese for reading and writing, and all were born, raised, and had received formal education in Hong Kong. Their age at the time of testing was 60 years or older (*M*_age_ = 64.42 years, *SD*_age_ = 2.99). The majority of the participants had attained a secondary level of education or lower (*n* = 43), while others had reached the university (*n* = 21) or postgraduate level (*n* = 12). All participants reported having normal or corrected-to-normal vision and none reported any history of psychological or neurological disorders.

All participants completed a Chinese vocabulary test (Tsang, 2025), which was shown to correlate with word recognition performance, comprehension accuracy, Chinese subject score in public examination, and self-rated Chinese proficiency. The scores of the current sample (*M*_vocab_ = 54.41, *SD*_vocab_ = 18.49) were comparable to those reported in other studies with this population (*M*_vocab_ = 54.29, *SD*_vocab_ = 19.29; Tsang et al., 2025). The study was approved by the Research Ethics Committee at Hong Kong Baptist University (REC/21–22/0042), and all participants provided written informed consent prior to participation.

### Materials

**Fig. 1:**
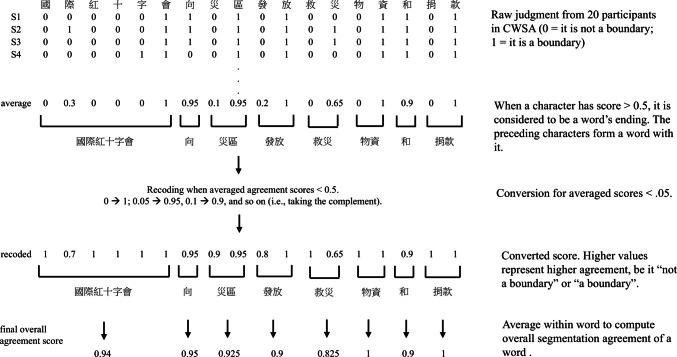
Defining word boundary and calculating segmentation agreement score. The sentence is translated as “The International Red Cross provided the disaster-affected region with aid materials and funds”

The stimuli consisted of 180 traditional Chinese sentences drawn from the Chinese Word Segmentation Agreement (CWSA) corpus (Tsang et al., 2025). The CWSA corpus provides segmentation judgments for 500 Chinese sentences (each by 20 native speakers), which we used to define word units and derive a segmentation agreement score for each word. This procedure involved several steps (see Fig. 1). First, for each potential boundary following a character, a rater’s judgment was coded as 0 (not a boundary) or 1 (a boundary). These were averaged across the 20 raters to produce a raw segmentation score for each potential boundary, ranging from 0 (complete agreement of no boundary) to 1 (complete agreement of boundary). Words were then defined based on this raw score: a character with a raw score of ≥0.5 was designated as a word-final character, grouping it with all preceding characters that had scores <0.5.

Next, to create a unified metric of segmentation ambiguity, the raw scores were transformed. Scores representing agreement against a boundary (raw scores < 0.5) were converted by taking their complements (0 became 1, 0.05 became 0.95, etc.). This resulted in a converted agreement score where 1 always represents perfect agreement (either for or against a boundary) and 0.5 represents maximum ambiguity. In this study, although we were interested in how word-level segmentation agreement might potentially modulate the effect of segmentation cues, it was also important to ensure that the segmentation was not completely ambiguous. Otherwise, the word segmentation condition could not be properly defined. With this consideration, we selected sentences where every potential boundary has a converted agreement score of 0.65 or above (i.e., agreed by at least 2/3 of the raters). Finally, for each word, the converted scores of its constituent boundaries were averaged to create a single word segmentation agreement score, reflecting how strongly the characters bind together as words.

The final set of 180 sentences contained 1,729 word tokens. Most of the words were two characters long (*n* = 1,131), followed by one-character (*n* = 407), and the rest were three characters or longer. The resulting word segmentation agreement scores for this set ranged from 0.65 to 1 (*M*_seg_ = 0.93 *SD*_seg_ = 0.07).

**Fig. 2:**
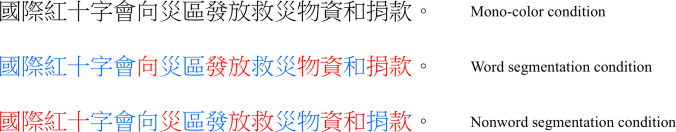
An illustration of the experimental stimuli in different conditions. (Color figure online)

These 180 sentences were presented in three experimental conditions (Fig. 2). In the monocolor condition, all characters were presented in black on a grey background. This is the baseline condition similar to normal everyday reading. In the word segmentation condition, all characters were grouped into words as defined above. Then, word boundary cues were provided by highlighting the words in alternative blue and red colors. Finally, invalid boundary cues were provided in the nonword segmentation condition, where color alternation marked nonword character strings, thereby breaking up the correct word units (e.g., by splitting longer words or shifting a boundary by one character). This condition was carefully controlled so that the number of colored segments did not differ significantly between the word (*M*_word_ = 9.61, *SD*_word_ = 1.68) and the nonword segmentation condition (*M*_nonword_ = 9.62, *SD*_nonword_ = 1.51), *t*(179) < 1, *p* >.5.

To ensure attentive reading, a yes/no comprehension question followed 42 of the 180 sentences (23.3%). The stimuli were distributed across three experimental lists using a Latin square design to prevent participants from viewing the same sentence more than once. Each list contained 60 sentences from each condition, resulting in 180 sentences in total. The three conditions were mixed and randomized during presentation. Participants were randomly assigned to one of the three lists. An additional 12 practice sentences were prepared to familiarize participants with the task and conditions.

This study followed previous studies (e.g., Pan et al., 2025) in adopting a within-subject manipulation of segmentation condition, such that each participant was exposed to both word and nonword segmentation cues. It may be argued that this procedure might weaken the benefits of word segmentation because the cues provided were not always reliable indicators of word boundaries. Indeed, Blythe et al. (2012) and Bai et al. (2013) adopted between-subject designs and observed more robust facilitation with word segmentation than Bai et al. (2008), which employed a within-subject design. However, these studies also differed in participant populations (children vs. adults), limiting strong conclusions. Moreover, it may be difficult for readers to strategically ignore the color cues even with a within-subject design because color is highly salient and strongly draws attention. Another relevant factor is the potential contrast difference in the monocolor and the two color-marked conditions. Although reduced contrast in the color-marked conditions may result in longer reading times because characters become less perceivable (Staub, 2020), yet this perceptual effect should influence word segmentation and nonword segmentation equally. If the two conditions led to facilitation and interference, respectively, compared with the baseline, a perception-based account may be insufficient. Given that previous studies adopting similar designs (Pan et al., 2025; Zhou et al., 2018) have successfully revealed facilitation in the word segmentation condition, we believe our results cannot be solely attributed to perceptual level factors or strategic processing.

### Apparatus

Eye movements were recorded with an EyeLink Portable Duo system (SR Research) running at 1000-Hz sampling rate using the head-stabilized tracking mode. The stimuli were presented on a 25-in. LCD monitor with a 1,920 × 1,080 resolution and a 240-Hz refresh rate. The participants were seated comfortably with their head position maintained by a chin-and-forehead rest at a distance of 60 cm from the monitor. While viewing was binocular, eye movements were recorded monocularly from the right eye. Each character was presented in DFKai-SB font and subtended approximately 1° of visual angle at a distance of 60 cm.

### Procedure

The participants were tested individually at a sound-attenuated laboratory. The experimental procedure and eye movement recording were controlled by Experiment Builder (SR Research). The participants’ gaze positions were calibrated with a 9-point grid using default parameters. After validation, a fixation point appeared on the left side of the screen for drift check. If the eye tracker identified a participant’s gaze on the fixation point, the point disappeared and a sentence appeared, with the center of the first character at the fixation point. Failure to detect a participant’s gaze on the fixation point triggered a recalibration. Each sentence was presented in a single line. The participants were instructed to read the sentences silently for comprehension, then fixate on a dot in the lower-right corner of the monitor, and finally press a keyboard button to signal trial completion. The participants were given 12 practice trials before the main experiment. Then, the 180 experimental sentences were presented randomly in one randomized block. Forty-two sentences were followed by an easy yes–no comprehension question. Participants provided responses by pressing corresponding keys on a keyboard. After removing two participants with low comprehension accuracy, the average accuracy was 91.24% (*SD* = 6.70%). Participants were also instructed to avoid excessive body and head movement during reading. However, they were permitted to take short breaks between trials, after which a recalibration was performed. After the reading experiment, the participants completed the vocabulary test.

### Analyses

Reading data was extracted with Data Viewer (SR Research). Sentence-level reading speed (RS; measured in character per minute) was obtained based on each whole sentence. Word-level eye-movement measures included first fixation location (FL; landing position of the first fixation on a word relative to word beginning, as measured in number of characters), first fixation duration (FFD; duration of the first fixation on a word during first-pass reading), gaze duration (GD; sum of fixation durations during first-pass reading of a word), and total reading time (TRT; sum of all fixations on a word including regressive fixations). Following Pan et al. (2025), analyses of eye-movement indices were restricted to two-character words because this is the most common word length in the sentences (over 65%) and single-character words tend to be skipped more often. First, three sentences were removed due to mismarking of boundaries. Second, trials were discarded if there were more than two blinks or tracker losses over the entire sentence. At the word level, data from sentence-initial and sentence-final words were removed, as were words containing blinks. Finally, words with FFD shorter than 60 ms or longer than 800 ms, and those with GD over 1,000 ms, were excluded. After screening, 42,786 data points (86.8% of 49,292 in the original set) remained for the eye-movement analyses.

Statistical analyses were performed using linear mixed-effects models, fitted with the *lme4* package (Bates et al., 2015) in R (Version 4.4.1). Separate models were constructed for five dependent variables: sentence-level RS, and four word-level eye-movement measures (FL, FFD, GD, and TRT). FFD, GD, and TRT were log-transformed prior to analysis. For the sentence-level model, the fixed effects involved presentation condition (monocolor, word segmentation, and nonword segmentation) and participants’ vocabulary score. For the word-level models, words’ segmentation agreement score was added as a third fixed effect. To examine whether vocabulary knowledge and segmentation agreement would modulate the effects of word boundary cues, we also included two hypothesis-driven two-way interactions in the word-level models—namely, the Condition × Vocabulary and Condition × Segmentation Agreement interactions. For all models, presentation condition was treatment-coded, using the monocolor condition as the baseline as it reflects normal reading. The continuous predictors, vocabulary knowledge and segmentation agreement scores, were centered and scaled by converting them into *z-*scores (across participants and words, respectively). All models included random intercepts for both participants and sentences. We initially attempted to fit a maximal random effects structure justified by the experimental design. However, to resolve convergence problems, the random structure was simplified. The final, best-fitting models for RS and TRT included random intercepts and by-participant random slope for presentation condition, while the other models included only random intercepts. The *p* values for the fixed effects were obtained using Satterthwaite approximation for degrees of freedom, as implemented in the *lmerTest* package (Kuznetsova et al., 2017). Data and code for analyses are available at OSF (https://osf.io/gn7tz/).

Given that multiple eye-movement indices were tested, the experiment-wise Type I error was inflated, and some corrections would be needed. Yet there is no agreed standard in the proper way of correction. Therefore, we adopted the rule-of-thumb criterion by von der Malsburg and Angele (2017) in interpreting effects that yielded consistent results in at least two dependent variables.

## Results

Table 1 shows the descriptive statistics in different conditions. The results of the linear mixed-effects models (Table 2) confirmed a robust interference from invalid nonword segmentation cues similar to Pan et al. (2025). As compared with the monocolor baseline, nonword segmentation slowed down sentence-level RS and increased word-level FFD, GD, and TRT (all *p* values <.001). This condition also resulted in FL nearer to the beginning of words (*p* =.012), a position less optimal for word recognition. In contrast, the word segmentation condition showed no significant differences from the monocolor baseline in all word-level eye movement measures (all *p* values >.1). Indeed, similar to the nonword condition, it was also associated with a significant decrease in RS (*p* <.001). We refrained from further elaborating on this interfering effect as it was only significant in RS.

**Table 1:** Reading indices in different conditions

	Monocolor	Word segmentation	Nonword segmentation
RS	402 (2.38)	392 (2.20)	351 (2.21)
FL	0.824 (0.00401)	0.818 (0.00401)	0.809 (0.00405)
FFD	245 (0.665)	246 (0.671)	250 (0.701)
GD	298 (1.13)	296 (1.10)	315 (1.25)
TRT	370 (1.76)	370 (1.79)	422 (2.17)

*Note.* Means (and standard errors of the means in parentheses) of different reading indices. RS = reading speed (in character per minute); FL = first fixation location (in character); FFD = first fixation duration (in ms); GD = gaze duration (in ms); TRT = total reading time (in ms)

**Table 2:** Summary of linear mixed-effects models

A. Reading speed	Est.	*SE*	*df*	*t*	*p*
(Intercept)	404.270	14.199	81.021	28.471	<.001
Vocab	17.530	14.290	73.812	1.227	.224
WS vs. MC	−11.060	2.922	72.430	−3.785	<.001
NS vs. MC	−54.707	2.967	72.539	−18.441	<.001
Vocab × (WS vs. MC)	−5.825	3.058	75.796	−1.905	.061
Vocab × (NS vs. MC)	−3.289	3.100	75.510	−1.061	.292
B. First fixation location	Est.	*SE*	*df*	*t*	*p*
(Intercept)	0.831	0.011	96.580	73.973	<.001
Vocab	0.012	0.011	87.290	1.046	.298
Seg	−0.003	0.005	25680	−0.622	.534
WS vs. MC	−0.005	0.006	42560	−0.832	.405
NS vs. MC	−0.015	0.006	42570	−2.523	.012
Vocab × (WS vs. MC)	−0.005	0.006	39920	−0.757	.449
Vocab × (NS vs. MC)	0.002	0.006	39630	0.350	.727
Seg × (WS vs. MC)	0.002	0.007	42520	0.306	.760
Seg × (NS vs. MC)	0.008	0.007	42520	1.134	.257
C. First fixation duration	Est.	*SE*	*df*	*t*	*p*
(Intercept)	5.456	0.014	81.960	379.589	<.001
Vocab	−0.039	0.015	77.950	−2.673	.009
Seg	−0.008	0.003	32480	−2.566	.010
WS vs. MC	0.000	0.004	42590	0.037	.971
NS vs. MC	0.016	0.004	42590	4.169	<.001
Vocab × (WS vs. MC)	0.010	0.004	41840	2.459	.014
Vocab × (NS vs. MC)	0.002	0.004	41730	0.579	.563
Seg × (WS vs. MC)	−0.003	0.004	42560	−0.822	.411
Seg × (NS vs. MC)	0.007	0.004	42560	1.739	.082
D. Gaze duration	Est.	*SE*	*df*	*t*	*p*
(Intercept)	5.599	0.017	89.520	329.640	<.001
Vocab	−0.062	0.017	78.770	−3.638	<.001
Seg	0.004	0.004	38400	0.948	.343
WS vs. MC	−0.005	0.005	42570	−1.048	.294
NS vs. MC	0.048	0.005	42570	9.387	<.001
Vocab × (WS vs. MC)	0.017	0.005	42570	3.342	.001
Vocab × (NS vs. MC)	0.005	0.005	42540	0.945	.345
Seg × (WS vs. MC)	−0.015	0.006	42550	−2.701	.007
Seg × (NS vs. MC)	0.002	0.006	42550	0.301	.764
E. Total reading time	Est.	*SE*	*df*	*t*	*p*
(Intercept)	5.772	0.025	93.460	227.384	<.001
Vocab	−0.048	0.025	73.260	−1.938	.057
Seg	0.007	0.005	42260	1.400	.161
WS vs. MC	−0.002	0.008	69.600	−0.233	.816
NS vs. MC	0.107	0.007	71.690	14.432	<.001
Vocab × (WS vs. MC)	0.018	0.009	69.890	2.051	.044
Vocab × (NS vs. MC)	0.004	0.008	71.360	0.490	.626
Seg × (WS vs. MC)	−0.013	0.007	42470	−1.995	.046
Seg × (NS vs. MC)	0.014	0.007	42490	2.130	.033

*Note.* Vocab = vocabulary score (*z*-score transformed); Seg = segmentation agreement score (*z*-score transformed); MC = monocolor condition; WS = word segmentation condition; NS = nonword segmentation condition

Participants’ vocabulary score was a significant predictor of word-level fixation durations. As expected, those with high vocabulary knowledge could recognize words more quickly, as revealed in the shorter FFD and GD (*p* values <.01). Finally, the words’ segmentation agreement scores only showed a main effect in FFD (*p* =.010). While this effect might be interesting, it was not stable enough to warrant further discussion.

**Fig. 3:**
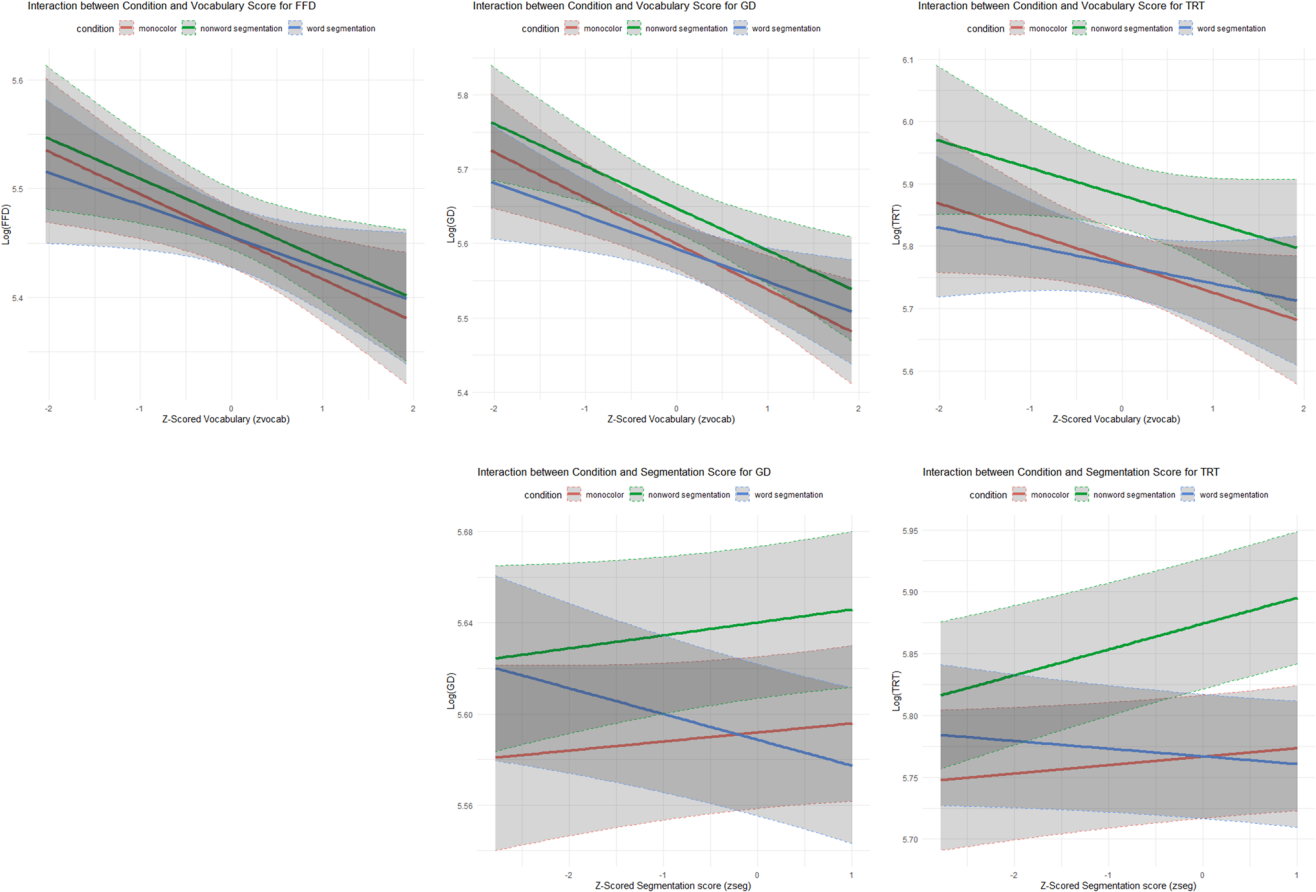
Interactions between vocabulary score/segmentation agreement and condition. (Color figure online)

Critically, the analyses also revealed significant interactions between the presentation condition (word segmentation vs. monocolor) and the vocabulary score for FFD, GD, and TRT, indicating that fixation durations increased in the word segmentation condition over the monocolor baseline as vocabulary scores rose (Table 2). In particular, only the readers with lower vocabulary scores (negative *z*-transformed values) exhibited shorter fixation durations in the word segmentation condition than the baseline. These patterns are visualized in Fig. 3, illustrating how vocabulary score modulates the effectiveness of word segmentation cues.

Similarly, interactions were found between the word segmentation versus monocolor contrast and *z*-transformed segmentation agreement score in GD and TRT, indicating that the benefits of word segmentation increased with higher segmentation agreement (Fig. 3). In contrast, when consensus about word boundaries is low, providing external cues interfered with reading, presumably because the cues may not always align with each reader’s segmentation.

## Discussion

Previous studies with simplified Chinese have shown that while nonword marking hampers reading, correct word boundary cues are beneficial (Pan et al., 2021, 2025; Zhou et al., 2018). This study extended previous works to older readers of traditional Chinese, who are characterized by a combination of age-related visual and cognitive declines alongside a lifetime of reading experience. Furthermore, the traditional Chinese script is visually more complex than simplified Chinese, which may place additional demands on the older readers. Two novel features of the present study stand out. At the item level, we employed sentences that varied in word boundary ambiguity, thereby extending the effect of word boundary marking to typical Chinese texts. At the participant level, we tested whether the benefits of marking word boundaries are modulated by individual differences in vocabulary knowledge. As such, the current study provides insights to word segmentation in Chinese reading.

Consistent with simplified Chinese, we observed a robust interference effect from nonword segmentation in traditional Chinese. In contrast, word segmentation did not produce a general, overarching benefit. However, this was qualified by a crucial interaction with a word’s segmentation agreement. A facilitative effect of word segmentation emerged in GD and TRT for words with high segmentation agreement. A low agreement implies that different readers tend to parse the character string differently. An externally imposed boundary can thus mismatch with a reader’s personal segmentation. In other words, to some participants, the color-marked character segments may be shorter or longer than their internally stored words, diluting or even reversing the benefit from boundary cues. Consistent with this idea, Pan et al. (2021) demonstrated that children who were better able to segment character strings according to linguistic standards benefited more from word boundary cues, presumably because their segmentation aligned better with the one imposed by the experimenters.

This interpretation helps reconcile our results with the clear facilitation reported in Pan et al. (2025), who used sentences containing words with high segmentation agreement. Our findings that the benefits of word segmentation cues may be specific to high-agreement words have both methodological and theoretical implications. From a methodological standpoint, this highlights the importance of considering segmentation agreement in testing the effectiveness of explicit boundary cues in Chinese reading. Depending on the exact research questions, one may need to measure and control for segmentation agreement in the materials, as failing to do so could mask or confound the effects of other manipulations. Theoretically, models of eye-movement control in Chinese and other unspaced scripts must account for the probabilistic nature of word segmentation. Individual judgments and idiosyncratic parsing of character strings are not simply noise, but an important component of the reading process that can override external visual cues.

The effect of providing word segmentation cues was also qualified by an interaction with readers’ vocabulary score, such that a benefit was observed only in readers with lower vocabulary knowledge. This result aligns with previous studies that have documented more robust benefits for other low proficiency readers, including children and L2 learners (Blythe et al., 2012; Pan et al., 2021; Zhou et al., 2020). Readers with strong vocabulary knowledge may have more extensive reading experience and are more likely to have developed robust, high-quality lexical representations (Perfetti, 2007). For them, word segmentation can rely on these internal representations and is relatively effortless. Then, external visual cues may be redundant and even introduce irregularities that disrupt their highly practiced and efficient reading routines. Supporting this view, Pan et al. (2014) examined eye movements of children with dyslexia and age-matched typically developing readers. Children with dyslexia not only exhibited longer fixations, but also directed their eye gaze closer to word beginnings, indicating a more effortful process of word segmentation, which can potentially be compensated with external cues.

The interference in the nonword segmentation conditions, particularly in FL, is clearly compatible with the flexible saccade planning model (Yan et al., 2010), which posits that Chinese readers segment words in the parafovea, and that selection of saccadic targets depends on the success of parafoveal segmentation. Our data indicates that misaligned character groupings at the perceptual level may disrupt readers’ word segmentation at the semantic level, which in turn interferes with saccade planning. In contrast, although the processing-based model denies the parafoveal segmentation process, it may explain the left-shifted FL in the nonword segmentation condition as a consequence of increased foveal processing load during prior fixations. The model also suggests that word segmentation is a by-product of word recognition, and any factor that facilitates foveal word recognition should facilitate foveal segmentation. From this perspective, readers can recognize words more rapidly and effortlessly in the word segmentation condition than in the monocolor segmentation condition, which saves cognitive resources for parafoveal processing of upcoming text in the former case and results in longer saccades and more right-shifted FL. The present results cannot definitively adjudicate between these two alternatives. Their primary difference is when segmentation occurs (during parafoveal vs. foveal word recognition). To disentangle these accounts, future research can employ a gaze-contingent boundary paradigm, as in Zhou et al. (2018). This method allows specific manipulation of segmentation cues in the parafovea before the eyes land on a word. Observing a similar interaction under these controlled parafoveal-only conditions would provide stronger support to flexible saccade planning.

The demonstration that explicit boundary cues are most beneficial for less proficient readers has important practical implications to educational and assistive technologies. Publishers, for instance, could incorporate such cues into Chinese reading materials designed for low-fluency readers, including typically developing children, as well as dyslexic and poor readers, while software applications could be developed to dynamically insert word boundaries into digital text. Furthermore, continuous exposure through such tools may mitigate the initial processing costs associated with an unfamiliar format, as observed in RS of the high fluency readers in this study, potentially enhancing the overall effectiveness of such cues over time. Once readers improve their lexical quality, they may read standard text fluently without such aids. This possibility should be tested in future studies. To summarize, this study shows that reading fluency and words’ segmentation agreement should be considered in understanding Chinese reading process and developing aids to improve reading performance. It enhances understanding of the interplay between readers’ experiences and the texts’ characteristics in reading, constraining the parameter spaces in computer simulation of Chinese reading.

One limitation of this study is the absence of a young reader group for comparison. While the effects of providing explicit boundary cues in Chinese reading has been relatively well-studied in young adults (Bai et al., 2008; Perea & Wang, 2017), the modulating roles of individual difference and segmentation agreement remain unclear. The possible effects of mixing word segmentation and nonword segmentation items, as well as visual contrast of the items, have not been tested. Further studies can incorporate these factors in the design for a more complete understanding of the role of word boundary cues in Chinese reading. Finally, a main effect of segmentation was found in FFD. Although it is consistent with Tsang et al. (2025) in suggesting that words with high segmentation agreement are read faster, the effect was not stably found in two or more eye movement indices, leading us to refrain from further discussion. Future work will be needed to investigate the temporal locus of segmentation agreement effect and whether a more robust effect can be found in young readers.

## Data Availability

The data and scripts for analyses are publicly available from this link (https://osf.io/gn7tz/) and the experiment was not preregistered.

## Code availability

See above.
